# Tetralogy of Fallot complicated by multiple cerebral abscesses in a child: a case report

**DOI:** 10.1186/s13256-024-04451-0

**Published:** 2024-03-28

**Authors:** Larrey Kasereka Kamabu, Franck Katembo Sikakulya, Louange Maha Kataka, Bives Nzanzu Mutume Vivalya, Hervé Monka Lekuya, Doomwin Oscar Deogratius Obiga, Juliet Nalwanga Sekabunga, Godfrey S. Bbosa

**Affiliations:** 1 Department of Surgery, Neurosurgery, College of Health Medicine, Makerere University, Mulago Upper Hill, Kampala, Uganda; 2 Faculty of Medicine, Université Catholique du Graben, Butembo, Democratic Republic of the Congo; 3 Department of Internal Medicine, Masereka General Hospital, North-Kivu, Democratic Republic of the Congo; 4 Department of Psychiatry and Mental Health, Kampala International University Western Campus, Ishaka, Uganda; 5 Department of Surgery, Kampala International University, Western Campus, Kampala, Uganda; 6 Directorate of Surgical Services, Neurosurgical Unit, Mulago National Referral Hospital, Kampala, Uganda; 7 Department of Pharmacology & Therapeutics, Makerere University College of Health Sciences, Kampala, Uganda

**Keywords:** Tetralogy of Fallot, Multiple cerebral abscesses, Child, Surgical drainage, And medical management

## Abstract

**Introduction:**

Brain abscesses are rare but potentially fatal condition and can be associated with cyanotic congenital heart disease of which 5–18.7% of these patients that develop cerebral abscess commonly have tetralogy of Fallot (TOF).

**Case presentation:**

We report a case of 3-year-old Muganda male that presented with convulsions, cyanosis and difficulty in breathing. The patient had a combination intervention of medical treatment and surgical drainage of the abscess. Post-operative Computerized tomography scan images and pre-operative brain Computerized tomography scans were compared. The multiple rings enhancing lesions were reduced in number and sizes. The largest measured ring was 44 × 22.5×16mm compared to the previous; 42 × 41×36mm. The mass effect had reduced from 16 mm to 7.5 mm. The periventricular hypodensities persisted. Findings showed radiological improvement with residual abscesses, subacute subdural hematoma and pneumocranium. The patient was treated with intravenous ceftriaxone 1 g OD for six weeks and he showed marked improvement and was discharged home after 3 months.

**Conclusion:**

A comprehensive strategy involving medications, surgical drainage, and early neurosurgical consultation is vital in treating brain abscesses in uncorrected TOF. Early identification of the pathogen, appropriate antibiotic therapy, and vigilant follow-up through clinical assessments and imaging are crucial, potentially spanning a 4–8-week treatment.

## Introduction

In the realm of pediatric medicine, complex and multifaceted cases continue to challenge healthcare practitioners, requiring a nuanced approach and innovative solutions. We describe unusual presentation of multiple parietooccipital brain abscesses in a 3-year-old male with tetralogy of Fallot (TOF) to highlight the importance and challenges of preoperative and postoperative diagnosis of multiple brain abscesses with unusual radiological features. Early detection and treatment can prevent complications such as cardiac, brain abscess, ventriculitis, obstructive hydrocephalus, and a wide range of coagulation defects that may increase the risk of anesthesia and surgery that can be used in the management of several brain abscesses due to TOF.

This case report delves into a particularly intricate and rare presentation: Tetralogy of Fallot, a congenital heart defect characterized by four distinct abnormalities, compounded by the unforeseen complication of multiple cerebral abscesses in a young child. Tetralogy of Fallot is a well-documented cardiac anomaly, but its convergence with cerebral abscesses represents a unique and exceptionally challenging clinical scenario. This case presents an opportunity to explore the diagnostic intricacies, therapeutic dilemmas, and interdisciplinary coordination required when managing such complex medical conditions in pediatric patients, underscoring the significance of an integrated approach to achieve the best possible outcome for these young individuals.

## Case presentation

A 3-year-old Muganda male, known child attending Mulago cardiac clinic, Kampala, Uganda and on propranolol was diagnosed with tetralogy of Fallot. He presented to the Emergency Department of Mulago National Referral Hospital (MNRH) with a one-week history of convulsions associated with generalized body weakness, easy fatigability, poor appetite and a nonproductive cough. Difficulty in breathing occurred after excessive crying. This was associated with worsening of the blue discoloration of the lips, palms and improved by the knee- chest position. Convulsion was generalized tonic–clonic GTC lasting about 10 min and associated with loss of consciousness (LOC) for almost 24 h. Child exhibited a stiffening of the muscles throughout the body and unusual rhythmic and jerking movements associated with repetitive and uncontrolled muscle contractions. No history of fever was reported.

Other systems were unremarkable. This was his third admission to the hospital. He has had recurrent episodes of difficulty in breathing since the age of 1½ years and was managed as pneumonia at MNRH with improvement. He was diagnosed with congenital heart disease on his second admission on the basis of echocardiography. His mother reported uneventful pregnancy, during which she attended Antenatal clinic (ANC) and serology for HIV was negative. She reported no history of cigarette smoking or alcohol consumption. The baby was delivered by spontaneous vertex delivery and cried immediately, with the birth weight of 2.5 kg. Neonatal period was uneventful. He is immunized up to date according to UNEPI vaccines schedule. Growth was normal during infancy with normal developmental milestones. Growth after 1 year was slow and had poor weight gain. He is the fourth born of 4 children; other siblings are well and normal. No familial history of cardiac disease, diabetes mellitus, asthma, sickle cell disease.

## Clinical findings

The physical examination revealed a Sick looking child, afebrile (T: 36.8c), no pallor, no jaundice, no edema, no lymphadenopathy, digital clubbing (grade 3). His anthropometry showed: weight 12 kg, height: 86 cm. Normal. GCS: 12/15, Eye opening: 4, Motor response: 5, Verbal response: 3. Neck soft, Kernig’s sign was negative. He had left facial nerve palsy grade 2. The muscle bulk were normal. Power, tone and reflexes exam are summarized (Table 1) and full blood count findings in the case were summarized (Table 2). Cardiovascular system: pulse rate: 125 bpm, regular, normal volume. Blood pressure: 96/60mmhg. The precordium was hyperactive. Heart sounds 1 and 2 were heard with an ejection systolic murmur best heard at the left upper sternal border. Respiratory system: RR: 35 bpm, SPO_2_: 55% RA. No respiratory distress, equal air entry, bronchovesicular breath sounds. Other systemic examinations were unremarkable.

**Table 1:** Power, tone and reflexes examination of the case

Motor exam	Response to motor exam			
	LUL	LLL	RUL	RLL
Power	Reduced	Reduced	Normal	Normal
Tone	hypertonia	Hypertonia	Normal	Normal
Reflexes	Hyper reflexia	Hyper reflexia	Normal	Normal

Key for abbreviations:

*LUL* left upper limb

*LLL* left upper limb

*RUL* right upper limb

*RLL* right lower limb

**Table 2:** Full blood count findings in the case

Hematological parameter	Hematological parameter follow up				
	23/11/22	5/12/22	23/1/23	19/2/23	02/3/23
WBC (X10^9^/l)	7.43	10.4	10.6	5.62	6.41
Lymphocytes	2.69	4.78	5.20	3.78	3.56
Neutrophils	4.17	3.83	4.39	1.46	2.42
HB (g/dl)		15.7	14.9	17.9	16
PLT	410	170	295	171	164
RFT/Serum electrolyte	N	N	N	N	N

*WBC* white blood cell; *HB* hemoglobin; *PLT* platelet; *RFT* renal functional test

## Diagnostic assessment

**Fig. 1:**
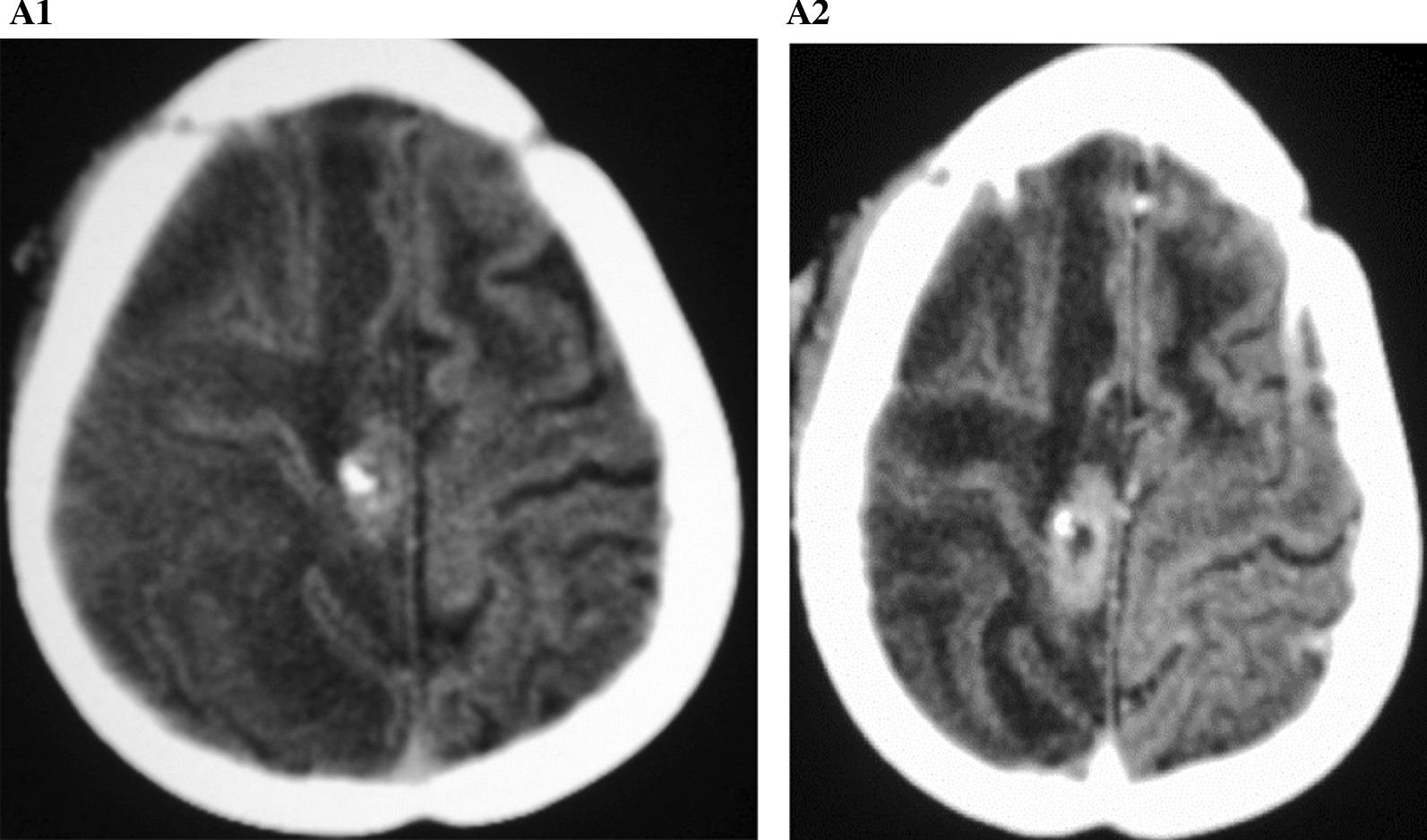
Computerized tomography scan findings of the case. **A1** None contrasted axial brain CT scan shows generalized hypodensity in the right hemisphere with no white–gray matter differentiation. A calcific lesion noted in the right perifissural region. **A2** Contrasted axial brain CT scan with no significant gyral enhancement

**Fig. 2:**
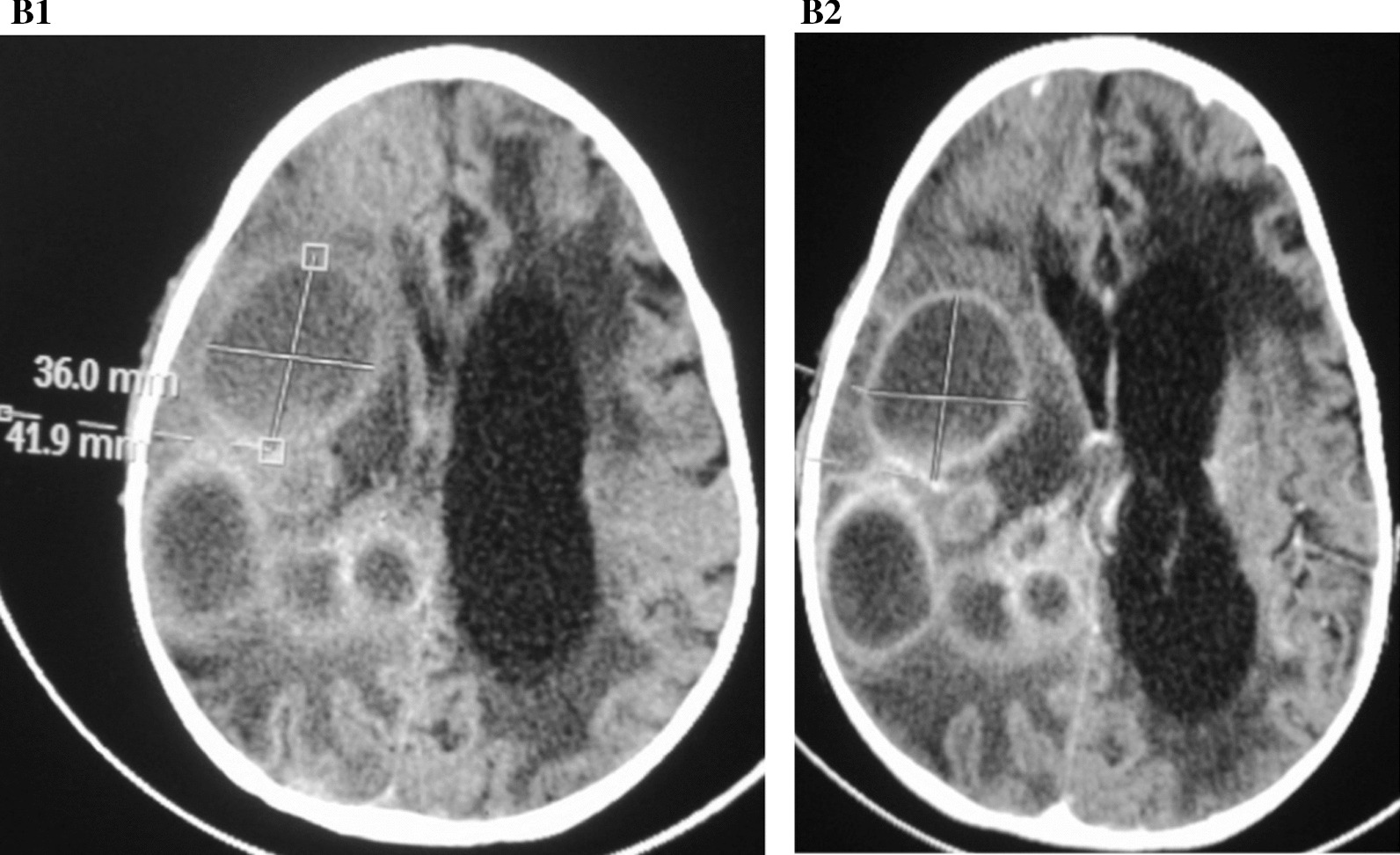
**B1** None contrasted axial scan shows: multiple well defined round lesions the biggest measures 42 × 41×36mm. **B2** Contrasted axial scan showing similar lesions with better definition of the margins. Left sided periventricular hypodensities of the lateral ventricle

**Fig. 3:**
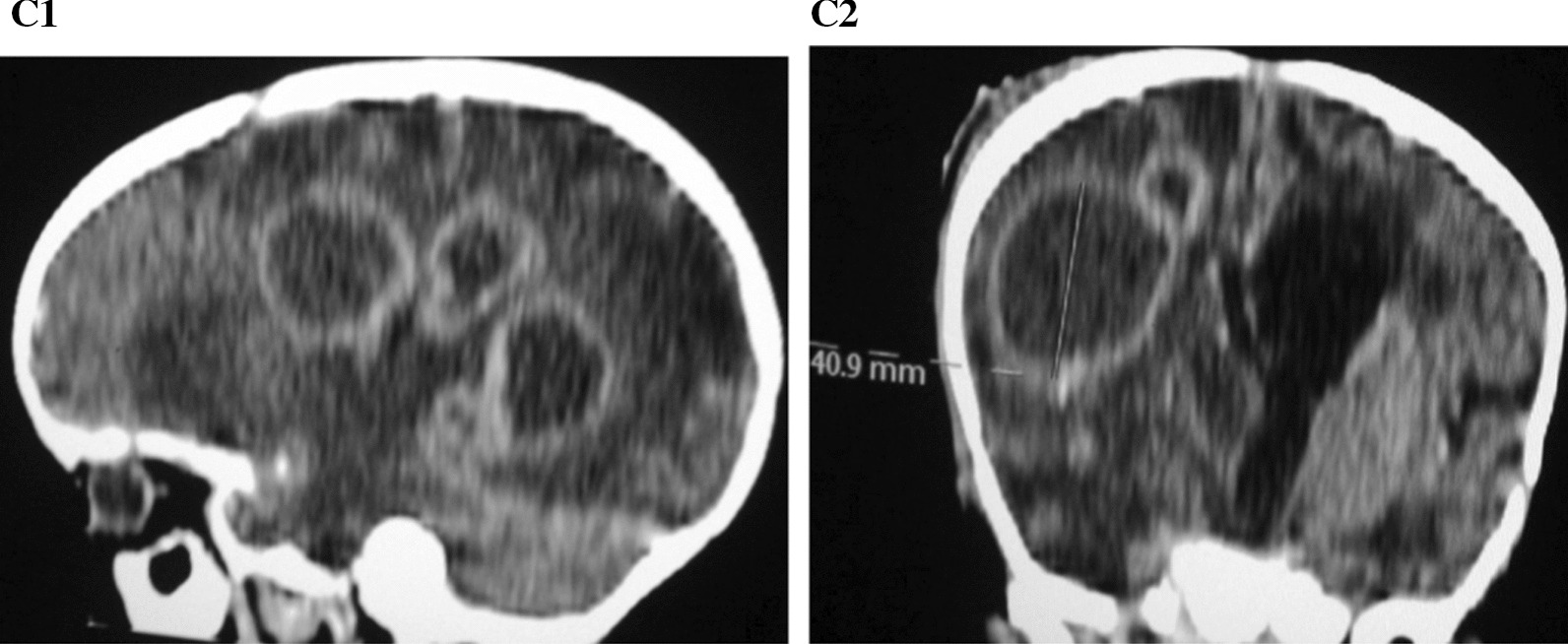
**C1** Sagittal reconstruction image showing multiple rings enhancing lesions in the right parietal and occipital lobes. The brain parenchyma is hypo attenuated. **C2** Coronal reconstruction image showing ring enhancing lesions in the right temporo-parietal lobes, compressed right lateral ventricle and dilated left lateral ventricle

**Fig. 4:**
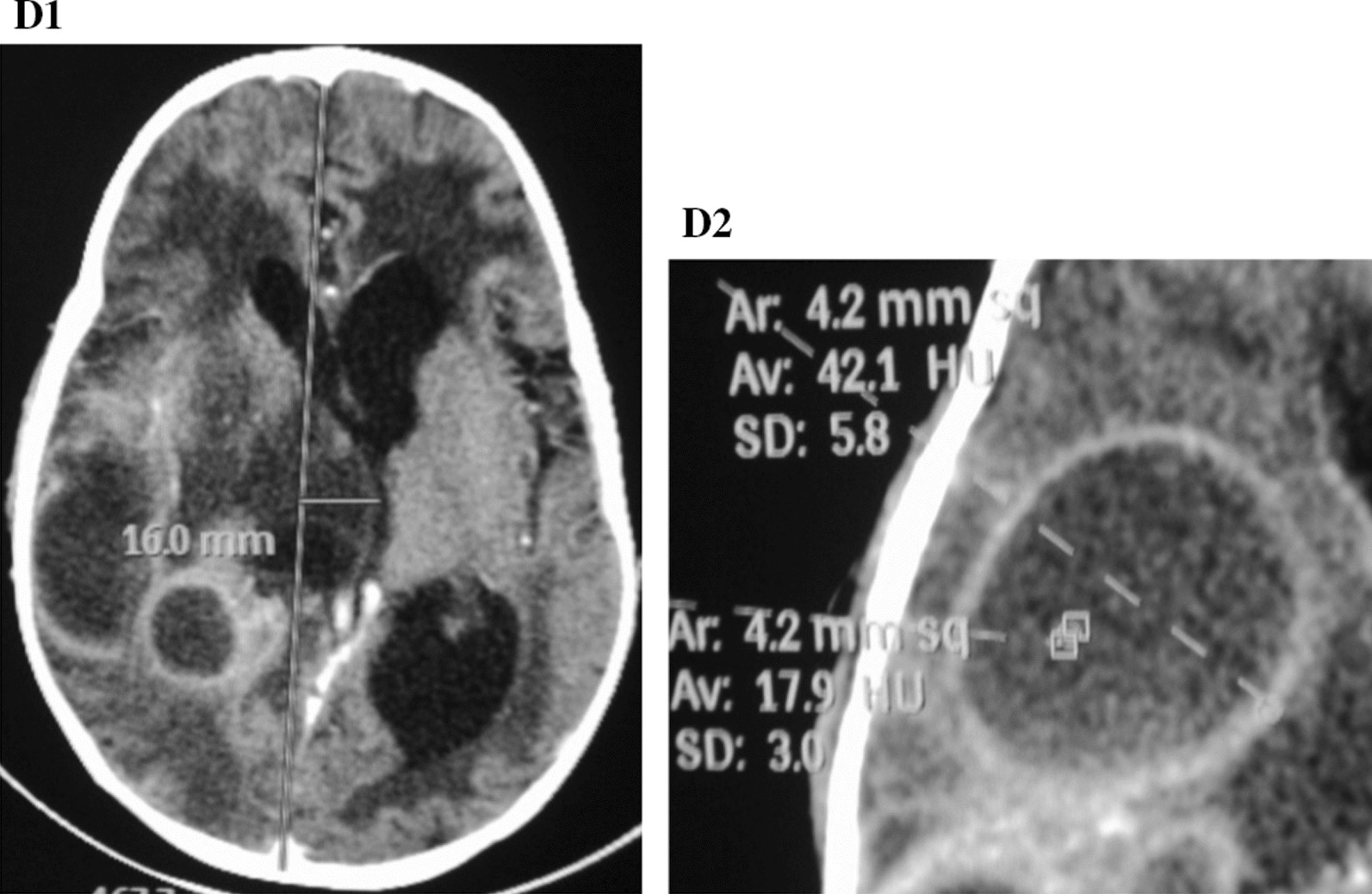
**D1** Axial brain computerized tomography scan showing a mass effect with a midline shift of 16 mm to the left. There is bilateral periventricular hypodensities. **D2** A close–up image showing a ring enhancement of the biggest lesion

**Fig. 5:**
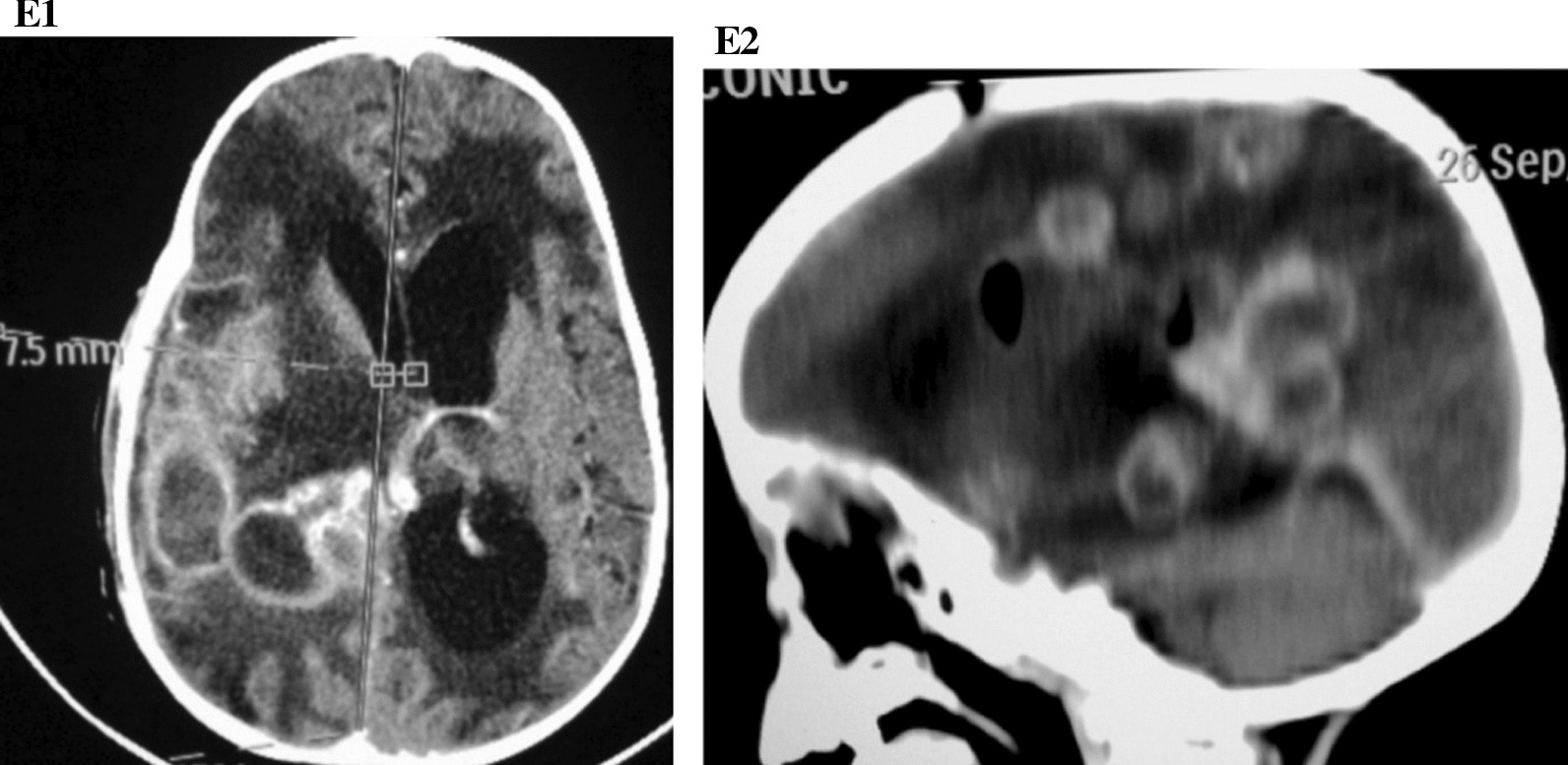
**E1** Axial brain computerized tomography scan showing reduction in the number and sizes of the ring enhancing lesions and mass effect. Subdural hematoma and persistence of the bilateral periventricular hypodensities. **E2** Sagittal reformat showing multiple residual ring enhancing lesions in the parietal and temporal lobes. Multiple intracranial pockets of air noted

An enhanced helical brain CT scans with sagittal and coronal reformats both pre- and post-operative were done. Preoperative scans (Figs. 1, 2, 3, 4, 5) showed multiple rings enhancing lesions in the right cerebral hemisphere. The largest ring measured 42 × 41×36mm, 18-42HU. The brain parenchyma on the right was also hypo-attenuated. There was a mass effect with a midline shift of 16 mm to the left. The left lateral ventricle was dilated whereas the right was compressed. Periventricular hypodensities were noted on both sides of the lateral ventricle. The skull bones appeared normal. Radiological diagnosis of multiple intracerebral abscesses with periventricular white matter disease, severely increased intracranial pressure with cerebral edema was made.

## Therapeutic intervention

A pre-operative resuscitation, antibiotic and antiseizures prophylaxis were given and followed by an emergency craniotomy was done and pus drained.

## Follow-up and outcomes

**Fig. 6:**
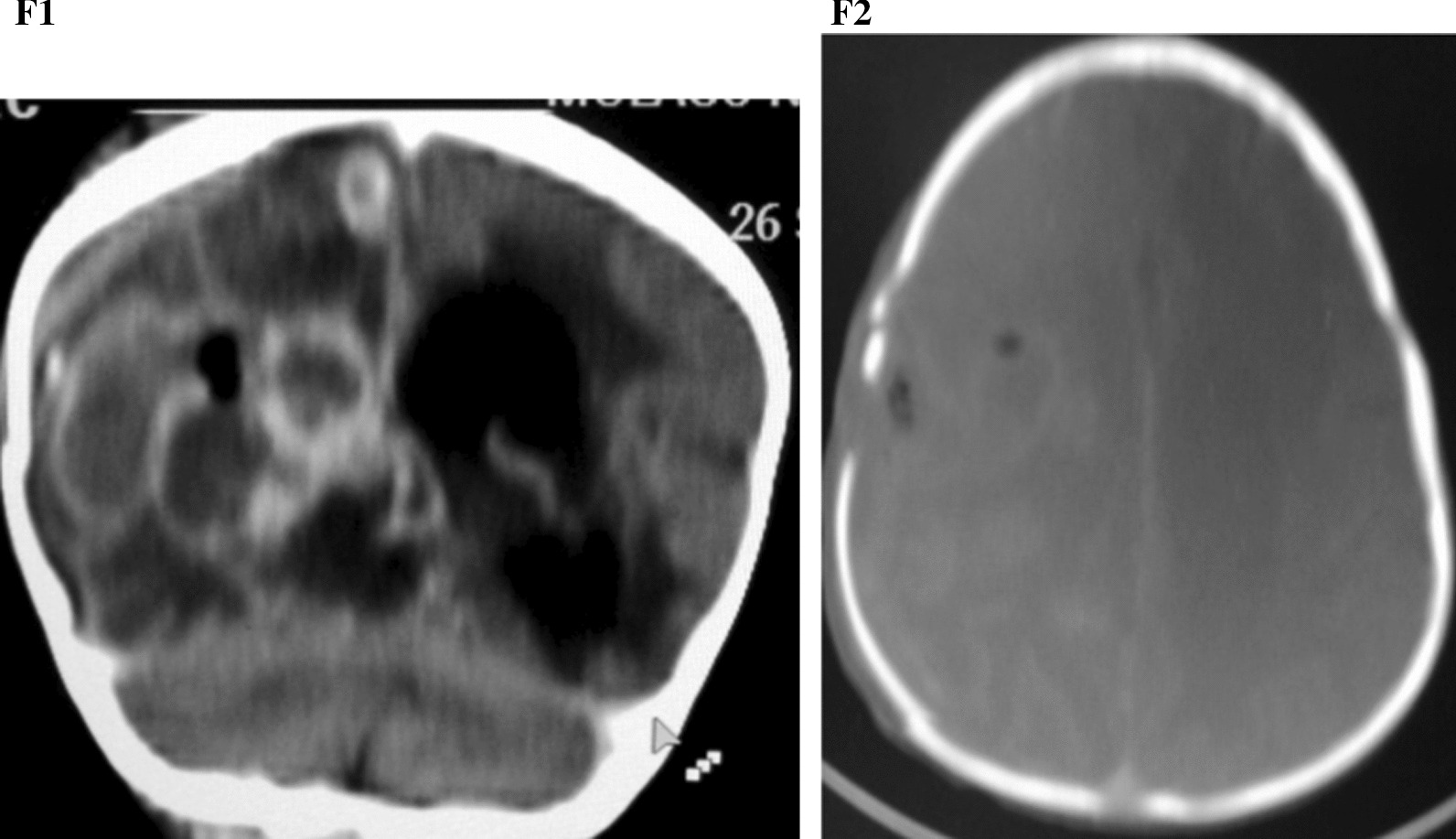
**F1** Coronal reformatted image showing residual ring enhancing lesions on the right cerebral hemisphere. Dilated left lateral ventricle. **F2** Bone reconstructed image showing a burr-hole defect of 10 mm in the right parietal region with intracranial pockets of air

The patient improved on therapeutic (surgical and medical) intervention with immediate return of consciousness and improvement of neurological symptoms. Post-operative CT scan images (Figs. 5, 6) and pre-operative brain CT scans were compared. The picture of the 3 year old boy following craniotomy and abscess drainage is presented in Fig. 7.The multiple rings enhancing lesions were reduced in number and sizes. The largest measured ring was 44 × 22.5×16mm compared to the previous; 42 × 41×36mm. The mass effect had reduced from 16 mm to 7.5 mm. The periventricular hypodensities persisted. In addition, multiple pockets of air were noted with a cranial defect of 10 mm with bone fragment noted on the right parietal bone with an associated concavo-convex hypodense lesion in the right temporoparietal region measuring 60 × 4 mm. Findings were in keeping with radiological improvement with residual abscesses and subacute subdural hematoma and pneumocranium.

On the third postoperative day, the patient was extubated after the craniotomy and pus was successfully drained. After the craniotomy, the patient's level of consciousness gradually returned to **15/15.** Patient was moved out of the intensive care unit and on post operative day 90, he was sent home.

## Discussion

The case of a 3-year-old male with Tetralogy of Fallot (TOF) who presented with multiple parietooccipital brain abscesses represents a remarkable medical challenge, emphasizing the critical significance of early detection and intervention in the context of this uncommon clinical scenario. TOF itself is a complex congenital heart condition characterized by four structural defects within the heart [1]. However, when coupled with the development of multiple brain abscesses, the situation becomes notably intricate [2].

Unusual radiological features complicate the preoperative and postoperative diagnosis of these abscesses [2]. Such atypical presentations can pose significant challenges for healthcare providers. Early recognition is crucial as these abscesses not only have the potential to exacerbate cardiac issues but can also lead to complications such as ventriculitis, obstructive hydrocephalus, and coagulation defects. These added complications can significantly heighten the risks associated with anesthesia and surgical intervention, making it imperative to manage the brain abscesses effectively to mitigate potential complications and ensure the best possible outcome for the patient.

In this case, we have explored the intricate diagnostic journey, the multifaceted therapeutic strategies employed, and the vital role of interdisciplinary collaboration in addressing this unique clinical confluence of TOF and multiple cerebral abscesses. By delving into the complexities of this case, we aim to underscore the necessity of early detection and tailored management approaches to prevent severe complications and optimize the care of pediatric patients with TOF and associated brain abscesses.

TOF was the sole risk factor in the instance described in this case. Besides from infections of the middle ear, mastoids, face, orbit, or scalp, other risk factors including penetrating wounds, comminuted fractures, intracranial operations, congenital lesions of the scalp and neck, and immune system abnormalities [3, 4]. With the exception of tetralogy of Fallot, this infant lacked all of these risk factors.

Patients with cyanotic congenital heart disease often possess not only cardiopulmonary risk but a wide variety of coagulation defect which enhance the risk of anesthesia and surgery. Empirical antibiotic treatment currently recommended in management of brain abscesses include Cefotaxime or ceftriaxone and an Nitroimidazole (Metronidazole, Tinidazole) based antibiotic, which should be changed according to susceptibly sensitivity profile of the causative organism. The suggested duration of treatment of brain abscesses is 6–8 weeks [5]. An alternative management is surgical drainage or a combination of the two especially in children with none resolving abscess after medical therapy. This patient had a combination of medical and surgical drainage of the abscess. He was treated with intravenous ceftriaxone 1 g OD for six weeks and he showed marked improvement.

The management of brain abscesses due to TOF requires a specialized center especially one that is involved in the management of congenital heart disease. The center should have the capability to evaluate and monitor patients with cyanotic heart disease [6]. The treatment of CCHD and the early identification of significant septal defects have benefited from significant medical improvements, particularly in surgery and interventional techniques, which have transformed the prognosis for these individuals [6] Children with CCHD require special attention from pediatric cardiologists, who must be properly incorporated into a multidisciplinary team of professionals caring for these cyanotic patients when they graduate from pediatric cardiology and following a suitable transition and transfer to adult care [7, 8].

Regular follow-up at a congenital heart disease center, closely collaborating with a family doctor and a community cardiologist; discussion of advance care and palliative care planning in these patients with limited life expectancy [6].

## Conclusion

In conclusion, the case of a 3-year-old child with Tetralogy of Fallot complicated by multiple cerebral abscesses highlights the intricate challenges in diagnosis and management of this rare clinical scenario. Early detection and intervention are paramount, given the potential for severe complications, including cardiac deterioration and neurological sequelae. This case underscores the critical importance of a multidisciplinary approach, emphasizing the need for vigilant monitoring, prompt treatment, and tailored care to ensure the best possible outcome for such complex pediatric patients.

## Data Availability

Not applicable.

## Abbreviations

GCS: Glasgow coma scale
MNRH: Mulago National Referral
LOC: Loss of consciousness
